# Effect of safranal, a constituent of saffron, on olanzapine (an atypical antipsychotic) induced metabolic disorders in rat

**DOI:** 10.22038/IJBMS.2019.13992

**Published:** 2019-12

**Authors:** Sara Malekzadeh, Mahmood Reza Heidari, Bibi Marjan Razavi, Maryam Rameshrad, Hossein Hosseinzadeh

**Affiliations:** 1 School of Pharmacy, Mashhad University of Medical Sciences, Mashhad, Iran; 2 Department of Pharmacodynamics and Toxicology, School of Pharmacy, Kerman University of Medical Sciences, Kerman, Iran; 3 Targeted Drug Delivery Research Center, Mashhad University of Medical Sciences, Mashhad, Iran; 4 Department of Pharmacodynamics and Toxicology, School of Pharmacy, Mashhad University of Medical Sciences, Mashhad, Iran; 5 Pharmaceutical Research Center, Pharmaceutical Technology Institute, Mashhad University of Medical Sciences, Mashhad, Iran\

**Keywords:** Crocus sativus, Metabolic syndrome, Obesity, Olanzapine, Safranal, Saffron

## Abstract

**Objective(s):**

Olanzapine, an atypical antipsychotic, causes weight gain and metabolic disorders in humans. Safranal, one of the active components of *Crocus sativus* (saffron), has been shown to have anti-obesity, lipid and blood pressure lowering and anti-diabetes effects. In this investigation, the effect of safranal on metabolic disorders induced by olanzapine was studied.

**Materials and Methods:**

Fourty-two female Wistar rats were divided into 7 groups of 6 animals. The two groups were selected as controls, which received olanzapine and safranal solvents, respectively. The third group treated by olanzapine 5 mg/kg. Groups 4, 5 and 6 treated by olanzapine 5 mg/kg plus safranal (2.5, 5 and 10 mg/kg) and the last group received safranal 10 mg/kg. The injections were performed intraperitoneally for 14 days and on the 15^th^ day the rats were killed and their serum were collected to measure metabolic factors including glucose, insulin, triglyceride, total cholesterol and HDL cholesterol. Leptin level in plasma was also measured. Mean systolic blood pressure was measured using tail cuff method at the end of study. The rats were weighed every other day and amount of food consumed was measured daily.

**Results:**

Olanzapine significantly elevated body weight, food intake, fasting blood glucose, TG, leptin, and mean systolic blood pressure (MSBP). It also significantly decreased HDL cholesterol blood level. Safranal significantly improved all these complications at three doses.

**Conclusion:**

Based on the results of this study, safranal is thought to be used as an effective combination in controlling metabolic complications caused by olanzapine.

## Introduction

Today, second generation antipsychotics (sGA) are the first line treatment for schizophrenia, because of the less extrapyramidal side effects, and more effectiveness on negative symptoms of schizophrenia. One of the drugs which is used widely in treatment of schizophrenia, bipolar disorder and some other psychotic disorders, is olanzapine. It has metabolic adverse effects such as weight gain, increase in adipose tissue mass, blood leptin, insulin levels, insulin resistance, type 2 diabetes, high blood pressure and disruption of fat profile (1, 2). Investigations have shown that olanzapine has the most risk of weight gain between other SGAs (3, 4). The incidence of obesity, which is considered as an important health problem has been increasing worldwide (5). The role of olanzapine and clozapine in the initiation or intensification of type 2 diabetes more than other antipsychotics has been established (6-8), and the risk of type 2 diabetes with olanzapine increases with elevating doses (9). To reduce the adverse effects of olanzapine, especially metabolic complications and weight gain, many studies have been carried out, and the effects of herbal drugs (10, 11) and chemical drugs (12-15) were investigated.

Because of safety, efficacy, cultural acceptability and lesser side effects of herbal medicine, recently, they are considered as a good candidate for the therapy of various diseases such as metabolic syndrome (16). These herbs and their active constituents include *Vitis vinifera* (17, 18), *Nigella sativa* (19), *Allium sativum* (20), *Rosmarinus officinalis *(21), *Crocus sativus* (16), *Persea americana *(22), *Cinnamomum verum* (23), *Camellia sinensis* (11), thymoquinone (19) and rutin (24). 

*Crocus sativus* L., which is known as saffron, is a perennial herb of the Iridaceae family (25, 26). Due to its powerful smell and natural yellow color, saffron is a remarkable medicinal herb and spice (26-28). Safranal, with systematic name: 2,6,6-trimethylcyclohexa-3,1-din-1-carboxaldehyde, is a volatile carboxaldehyde compound (29), it is easily obtained by deglycosilation of picocrocin , one of the main organoleptic constituents of saffron (25). Safranal exhibited useful effects on the central nervous system (30) and respiratory system (31, 32). This compound is a potent antioxidant that has many useful effects due to scavenging of free radicals. These effects include regulation of the antioxidant properties of myocardium, modulation of the level of nitrotyrosin and cardioprotective markers (LDH and CK-MB) (33), and it can act as a potent anti-apoptotic chemical agent (32). This compound could reduce blood pressure with a decrease in heart rate and vascular relaxation (34). Safranal also has beneficial metabolic effects, these effects include decrease of fasting blood glucose (FBS), and Hb_A1c _serum level, increase sensitivity to insulin (35) and it also improves the altered serum lipid profile in diabetic patients (36-38). 

According to this fact that many schizophrenic patients receiving olanzapine are at increased risk of weight gain and other adverse effects, and safranal is also known to be effective in modulating and regulating these metabolic disorders, this study evaluated the effects of safranal on metabolic disorders induced by olanzapine in female rats.

## Materials and Methods

***Animals***

To evaluate the effects of safranal on metabolic complications induced by olanzapine, 42 female Wistar rats weighing 170 to 200 g from the Buali Institute and the Animal Department of the Mashhad Faculty of Pharmacy were obtained. The age of the animals was 6 to 8 weeks. Animals were exposed to a temperature of 21+2 ^°^C and in 12 hr period of darkness and brightness. Two rats were kept in a box during the study. All animals experiment were conducted according to the rules of the ethics committee of Mashhad University of Medical Sciences (project code: 941401).

***Study design***

To evaluate the effects of safranal on metabolic complications induced by olanzapine, 42 female Wistar rats were divided into 7 groups of 6. Group 1: rats receiving olanzapine solvent (injectable normal saline with a small amount of glacial acetic acid); group 2: rats receiving safranal solvent (injectable normal saline with a small amount of DMSO and a drop of tween 80) once daily for 14 days; group 3: rats receiving 5 mg/kg of olanzapine once daily for 14 days via intraperitoneal injection (39); group 4: rats receiving 5 mg/kg olanzapine once daily for 14 days via intraperitoneal injection with safranal (2.5 mg/kg/day) for 14 days (32); group 5: rats receiving olanzapine 5 mg/kg once daily for 14 days via intraperitoneal injection with safranal (5 mg/kg/day) for 14 days (34); group 6: rats receiving olanzapine 5 mg/kg once daily for 14 days via intraperitoneal injection with safranal (10 mg/kg/day) for 14 days (34); group 7: rats receiving 10 mg/kg of safranal once daily for 14 days via intraperitoneal injection.

***Plasma metabolic factors***

On the 15^th^ day of study, the rats were killed under fasting condition and their serum was isolated using a 20 min centrifuge at 4500 rpm, sent to the laboratory for measurement of metabolic factors including glucose, insulin, triglyceride, total cholesterol and HDL cholesterol. Plasma leptin concentration was analyzed using the Abcam-Cambridge (uk) ELISA kit.

***Food intake and body weight***

Food intake was calculated daily by measuring the difference in the amount of food placed in the hopper and remaining amount at the end of the 24 hrs. Body weight was measured every other day during the study.

***Systolic blood pressure (SBP)***

At the end of study, SBP was measured by non-invasive tail cuff method (31). The blood pressure was measured from tail vein using NIBP controller. SBP was measured 5 times and the mean SBP was reported.

***Statistical analysis***

One-way and two-way ANOVA and Post test Tukey-Kramer tests were utilized to compare the different groups. *P<*0.05 was considered statistically significant. Results were expressed as mean±SEM.

## Results

We had two solvent control groups, but because the results of these two groups were not statistically significant, just one of them was used.

***Weight changes***

**Figure 1:**
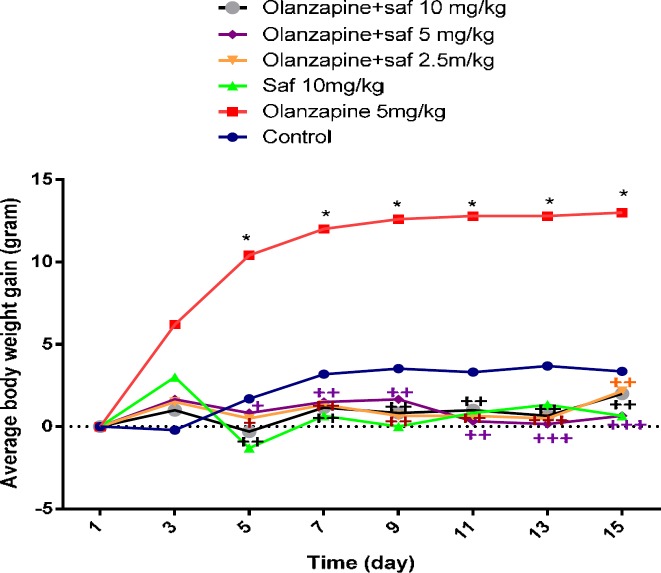
Investigating the effect of olanzapine 5 mg/kg with safranal and alone on daily weight changes in female rats during 14 days of intraperitoneal administration. For statistical comparison, two-way ANOVA and Tukey-Kramer *Post hoc* test were used. Comparison with control (*) and comparison with olanzapine (+); *P<*0.05(+,*), and *P<*0.01(++), and *P<*0.001 (+++) (saf: safranal, olan: olanzapine). N=6

The mean weight changes in the olanzapine group, 5 mg/kg alone (grope 3), were significantly higher than the control group from the fifth day until the last day of the study (*P<*0.05). Safranal was able to significantly decrease the weight gain induced by olanzapine in all three doses of 2.5, 5 and 10 mg/kg.The mean weight in the group received olanzapine alone increased by 6.48% at the end of the 14-day administration compared to control group, this weight gain in the groups receiving 2.5, 5 and 10 mg/kg of safranal plus olanzapine was 4.04%, 3.76% and 3.51%, respectively compared to olanzapine group (Figure 1).

***Food intake***

**Figure 2:**
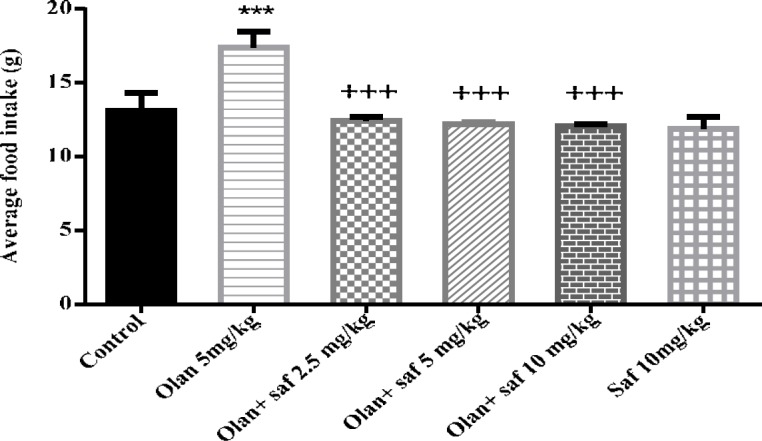
Investigating the effect of olanzapine 5 mg/kg with safranal and alone on food intake in female rats during 14 days of intraperitoneal administration. Values are displayed as SEM+Mean. For statistical comparison, one-way ANOVA and Tukey-Kramer test were used. Comparison with control (*) and comparison with olanzapine (+); *P<*0.001 (+++ , ***) (saf: safranal, olan: olanzapine)

Average food intake (g) in the olanzapine group, was significantly higher than the control group (*P<*0.001). The amount of food intake in safranal group alone was not significantly different from that of the control group. In this study, safranal was able to significantly preventing of increase the food intake induced by olanzapine) 5 mg /kg( in all three doses of 2.5, 5 and 10 mg/kg (*P<*0.001). Mean food intake in safranal groups at 2.5, 5 and 10 mg/kg plus olanzapine 5 mg/kg compared to the olanzapine group was decreased 28.55%, 29.65% and 30.40% respectively (Figure 2).

***Mean systolic blood pressure (MSBP)***

MSBP in the olanzapine group (5 mg/kg), was significantly higher than the control group (*P<*0.001). MSBP in safranal group alone was not significantly different from that of the control group (group 1). Also, in all three groups receiving olanzapine plus safranal (2.5, 5 and 10 mg/kg), there was a significant decrease in MSBP compared to the olanzapine group. 

**Figure 3:**
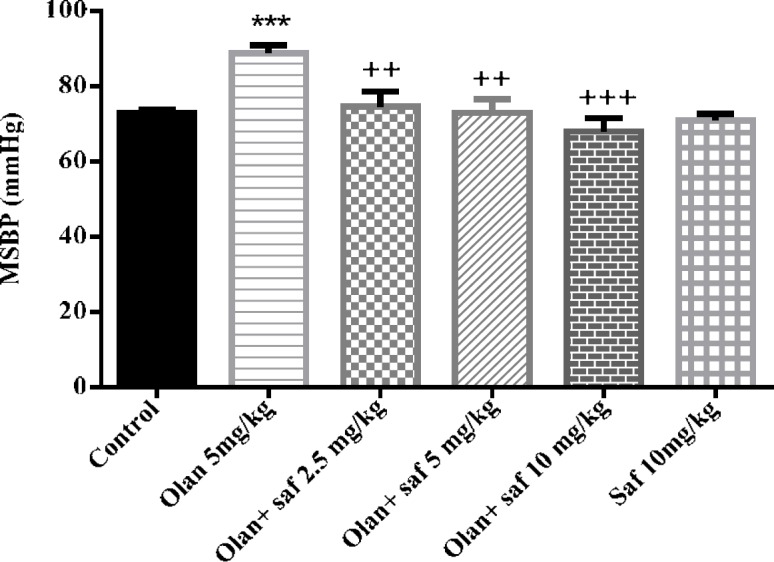
Investigating the effect of olanzapine 5 mg / kg with safranal and alone on mean systolic blood pressure (MSBP) changes in female rats during 14 days of intraperitoneal administration. Values are displayed as SEM+Mean. For statistical comparison, one-way ANOVA and Tukey-Kramer test were used. Comparison with control (*) and comparison with olanzapine (+); *P<*0.01(++) and *P<*0.001 (+++ , ***) (saf: safranal, olan: olanzapine)

The results showed that MSBP in group receiving 5 mg/kg olanzapine increased 24.71% compared to the control group; and the amount of reduction in MSBP in group receiving olanzapine plus safranal in doses of 2.5, 5 and 10 mg/kg compared to the group receiving olanzapine alone were 15.96%, 17.77%, and 23.32 mg, respectively (Figure 3).

***Fasting blood glucose (FBS)***

**Figure 4:**
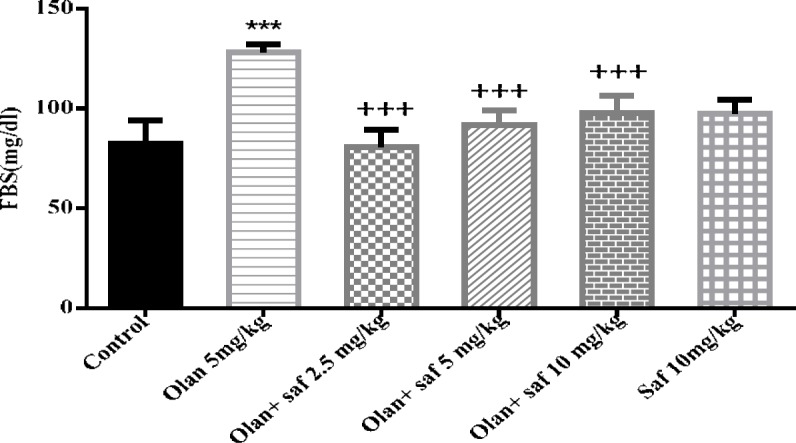
Investigating the effect of olanzapine 5 mg/kg with safranal and alone on fasting blood sugar (FBS) in female rats during 14 days of intraperitoneal administration. Values are displayed as SEM+Mean. For statistical comparison, one-way ANOVA and Tukey-Kramer test were used. Comparison with control (*) and comparison with olanzapine (+); *P<*0.001 (+++ , ***) (saf: safranal, olan: olanzapine)

Fasting blood glucose level in group receiving olanzapine showed a significant increase compared to the control group (*P<*0.001). FBS did not show any significant difference in safranal group alone compared to control group. All three groups receiving olanzapine plus safranal (2.5, 5 and 10 mg/kg) showed a significant decrease in FBS levels compared to the group receiving olanzapine alone (*P<*0.001). The results of this study showed a decrease of 37.41%, 28.57% and 23.79% in FBS levels in groups received olanzapine plus safranal (2.5, 5 and 10 mg/kg), respectively, compared to the group receiving olanzapine (Figure 4).

***Insulin serum level***

**Figure 5:**
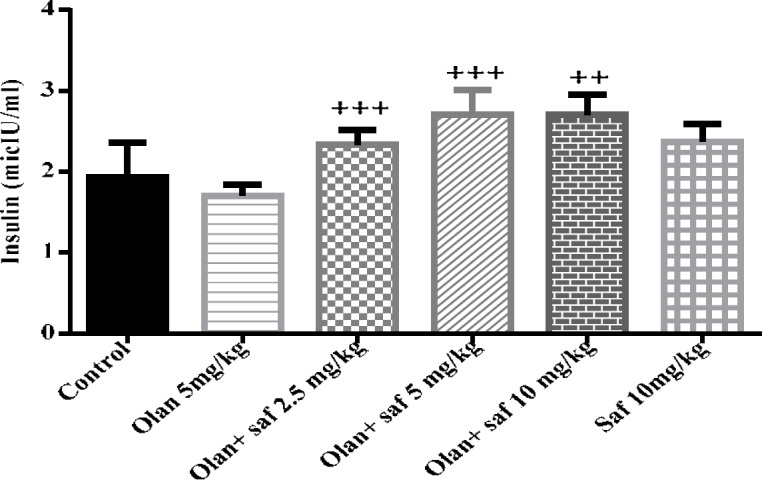
Investigating the effect of olanzapine 5 mg/kg with safranal and alone on insulin serum level in female rats during 14 days of intraperitoneal administration. Values are displayed as SEM+Mean. For statistical comparison, one-way ANOVA and Tukey-Kramer test were used. Comparison with control (*) and comparison with olanzapine (+); *P<*0.001 (+++ , ***) (saf: safranal, olan: olanzapine)

Insulin serum level in group receiving olanzapine showed 1.91% decrease compared to the control group, but it was not significant. In insulin serum level any significant difference in safranal group alone compared to the control group was not observed. The level of insulin was increased significantly in all three groups receiving olanzapine plus safranal (2.5, 5 and 10 mg/kg) *P<* 0.001, *P<*0.001 and *P<*0.01; respectively) (Figure 5).

***Leptin serum level***

**Figure 6:**
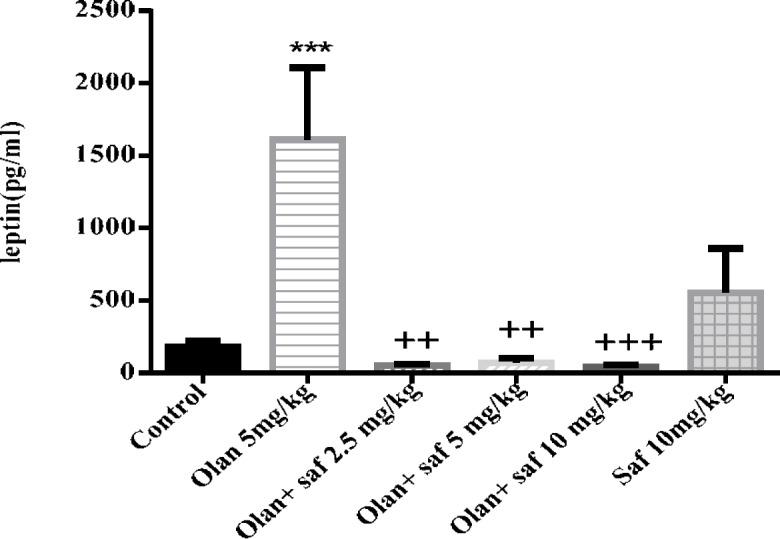
Investigating the effect of olanzapine 5 mg/kg with safranal and alone on leptin serum level in female rats during 14 days of intraperitoneal administration. Values are displayed as SEM+Mean. For statistical comparison, one-way ANOVA and Tukey-Kramer test were used. Comparison with control (*) and comparison with olanzapine (+); *P<*0.01(++) and P <0.001 (+++, ***) (saf: safranal, olan: olanzapine)

Plasma leptin level was significantly increased in olanzapine group compared to the control group (*P<*0.001). In all three groups receiving olanzapine plus safranal (2.5, 5 and 10 mg/kg), there was a significant decrease in leptin level compared to the olanzapine group (*P<*0.01, *P<*0.01 and *P<*0.001, respectively) (Figure 6).

***Triglyceride serum level***

**Figure 7:**
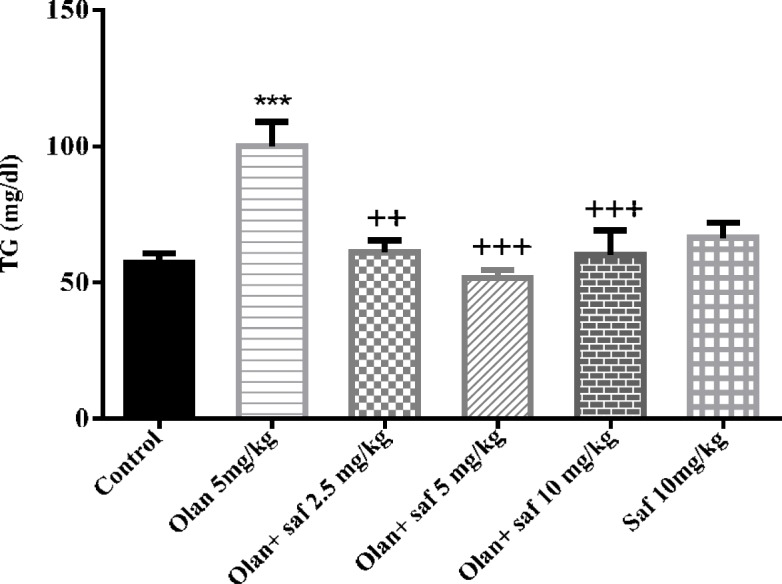
Investigating the effect of olanzapine 5 mg/kg with safranal and alone on triglyceride serum level in female rats during 14 days of intraperitoneal administration. Values are displayed as SEM+Mean. For statistical comparison, one-way ANOVA and Tukey-Kramer test were used. Comparison with control (*) and comparison with olanzapine (+); *P<*0.001 (+++ , ***) (saf: safranal, olan: olanzapine)

The level of triglyceride in the olanzapine group, was significantly higher than the control group (*P<*0.001). The level of triglyceride in the safranal group alone did not show any significant difference compared to the control group. Also, in all three groups receiving olanzapine plus safranal (2.5, 5 and 10 mg/kg), there was a significant decrease in triglyceride level compared to olanzapine (*P<*01, *P<*0.001 and *P<*0.001).Increasing triglyceride level in the olanzapine group compared to the control group was 77.51%. Reduced triglyceride level in the olanzapine group plus safranal (2.5, 5 and 10 mg/kg) compared to the olanzapine group was 38.8%, 48.33% and 39.83%, respectively (Figure 7).

**Figure 8:**
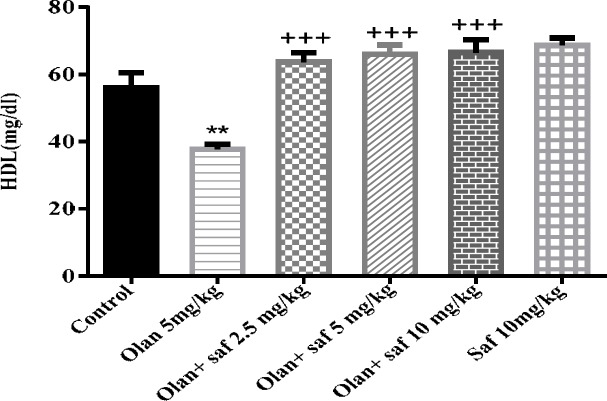
Investigating the effect of olanzapine 5 mg/kg with safranal and alone on HDL level in female rats during 14 days of intraperitoneal administration. Values are displayed as SEM+Mean. For statistical comparison, one-way ANOVA and Tukey-Kramer test were used. Comparison with control (*) and comparison with olanzapine (+); *P<*0.01(**) and *P<*0.001 (+++) (saf: safranal, olan: olanzapine)

**Figure 9:**
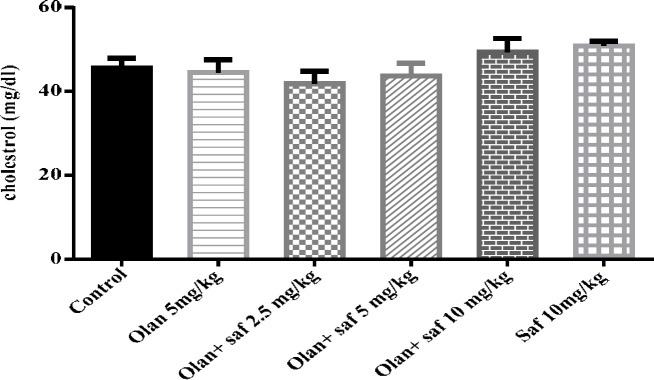
Investigating the effect of olanzapine 5 mg/kg with safranal and alone on cholesterol in female rats during 14 days of intraperitoneal administration. Values are displayed as SEM+ Mean. For statistical comparison, one-way ANOVA and Tukey-Kramer test were used

***HDL-C*** ***serum level***

HDL-C level in the olanzapine group was significantly lower than the control group (*P<*0.01). HDL-C did not show a significant difference in safranal group alone compared to the control group. All three groups receiving olanzapine plus safranal (2.5, 5 and 10 mg/kg) showed a significant increase in HDL-C in comparison with the olanzapine group (*P<*0.001).The results of this study showed an increase of 68.88%, 75% and 76.37% in HDL-C in groups received olanzapine plus safranal (2.5, 5 and 10 mg/kg), respectively, compared to the group receiving olanzapine (Figure 8).

***Total cholesterol serum level***

Serum levels of cholesterol in all tested groups were within a range and no significant difference was observed.

## Discussion

Safranal was able to prevent the increase of food intake, FBG, triglyceride, serum levels of leptin and MSBP and also reduced HDL-C induced by olanzapine in all three doses (2.5, 5 and 10 mg/kg). Safranal also significantly increased insulin serum levels in all three doses compared to those receiving olanzapine. The level of cholesterol was not affected by olanzapine. 

In the present study, like many other animal studies (40-42), administration of olanzapine increased food intake and weight gain significantly in female rats (32.80% and 6.48% respectively). Olanzapine also increases the weight and appetite in human studies (3, 4, 43-45). Increased hunger with a decrease in satiety has been reported consistently with the administration of olanzapine (45). Despite the introduction of several mechanisms, the precise mechanisms of olanzapine induced weight gain have not yet been determined, but in addition to increased appetite (40, 46, 47); insulin resistance (48), and increased serum leptin, are proposed (49-51). 

Leptin is one of the cytokine-like molecules synthesized from adipose tissue, which is actively transmitted to the hypothalamus, where its activity restricts food intake (52). It also directly affects the secretion of insulin (53). Therefore, leptin has been investigated for its association with changes in body weight and glucose metabolism during treatment with various anti-psychotic drugs (54). In previous studies, increase in the level of leptin has been reported in patients receiving olanzapine (49, 51, 55). In previous studies on saffron, authors determined that this plant reduces appetite (56, 57), decreases insulin resistance (58, 59), and reduces serum leptin levels (60). The results of measurement of food intake, serum level of insulin and leptin in this study also confirmed this. In previous studies, the role of saffron extract was mostly investigated. In this study, the effects of pure safranal as a major part of saffron extract were studied, which showed that safranal prevented olanzapine induced weight gain, which could be due to reduce appetite, decrease serum leptin levels, and increase insulin sensitivity.

Another metabolic complication caused by olanzapine is the initiation or intensification of type 2 diabetes (7, 8). Diabetes can be caused by impaired glucose tolerance and decreased insulin secretion from beta cells of the pancreas, due to the antagonistic effects of olanzapine. Studies on dog have shown that beta cell regression for insulin resistance may be reduced or even eliminated in the presence of olanzapine (48). This lack of increased insulin secretion can facilitate hyperglycemia (6). Saffron is used in diabetes because it has potent antioxidant effects (59, 61). The antioxidant effects reduce the levels of active oxygen species leading to the increase in insulin secretion from pancreatic beta cells, hypoglycemic effects, and increased tissue hypersensitivity to insulin (59). Safranal reduces inflammation of the pancreas and plasma and also decreases the oxidative stress of type 2 diabetes in plasma and pancreas by reducing TNF-α and IL-β levels (62).

The common clinical side effects of olanzapine are its effects on fat profile. In clinical and basic studies, the harmful effects of this drug on the fat profile have been proven (3, 63). One study found that olanzapine increased triglycerides and LDL, and the authors believed that triglyceride increases may be due to a reduction in the metabolic cleansing of triglycerides, or equivalent to an increase in liver lipogenesis (64). Saffron consumption can improve fat profile using antioxidant mechanisms (37). Saffron also increases the amount of fatty stools (65). It also selectively inhibits the activity of the pancreatic lipase as a competitive inhibitor (36). Safranal could have beneficial effects on altered lipid profile with olanzapine in rat and adjusted serum triglyceride and HDL levels. This result was obtained in previous studies by aqueous extract of saffron (36, 37) and we proved in this study that safranal could also have this effect.

Previous studies confirmed that saffron consumption reduced the high blood pressure in humans and rodents (66, 67). The antioxidant, diuretic and vasodilator effects are the three main mechanisms of anti-hypertensive effect of saffron (34). Recently, it has been shown that administering safranal for 5 weeks at 1, 2, 4 mg/kg/day, dose dependently decreases the systolic blood pressure in hypertensive rats receiving desoxycorticosterone acetate (DOCA) salts, but it does not change the blood pressure in rats with normal blood pressure (68). Similar to the tested rats in the present study, the mean systolic blood pressure increased in most patients receiving olanzapine (69). 

## Conclusion

According to the results of this study, safranal was effective to alleviate different metabolic disorders induced by olanzapine such as obesity, hyperglycemia, hypertriglyceridemia and hypertension. Therefore, it can be used as an effective combination in controlling the metabolic effects of olanzapine as a preventive treatment with olanzapine.
